# *Toxoplasma* Effector GRA15-Dependent Suppression of IFN-γ-Induced Antiparasitic Response in Human Neurons

**DOI:** 10.3389/fcimb.2019.00140

**Published:** 2019-05-01

**Authors:** Hironori Bando, Youngae Lee, Naoya Sakaguchi, Ariel Pradipta, Ryoma Sakamoto, Shun Tanaka, Ji Su Ma, Miwa Sasai, Masahiro Yamamoto

**Affiliations:** ^1^Department of Immunoparasitology, Research Institute for Microbial Diseases, Suita, Japan; ^2^Laboratory of Immunoparasitology, WPI Immunology Frontier Research Center, Osaka University, Osaka, Japan

**Keywords:** IFN-γ, IDO1, GRA15, human, neuron, toxoplasmosis, *Toxoplasma gondii*

## Abstract

*Toxoplasma gondii* is an important human and animal pathogen that causes life-threatening toxoplasmosis. The host immune system produces interferon-γ (IFN-γ) to inhibit *T. gondii* proliferation. IFN-γ-inducible indole-2,3-dioxygenase 1 (IDO1), which mediates tryptophan degradation, has a major role in anti-*T. gondii* immune responses in various human cells. In response to the host's immune system, *T. gondii* secretes many virulence molecules into the host cells to suppress IFN-γ-dependent antiparasitic immune responses. The GRA15-induced proparasitic mechanism for suppressing IDO1-dependent immune responses has previously been tested only in human hepatocyte and monocyte co-cultures. Thus, whether human cells other than hepatocytes contain this virulence mechanism remains unclear. Here, we show that the GRA15-dependent virulence mechanism for suppressing the IDO1-dependent anti-*T. gondii* response operates in human neuronal cell lines and primary human neurons. Analysis of various human cell lines revealed that IL-1β-induced iNOS-dependent reduction of IDO1 mRNA expression occurred in brain cell lines (A172; glioblastoma, IMR-32; neuroblastoma, and T98G; glioblastoma) and liver cell lines (Huh7 and HepG2), but not in other cell lines. Moreover, co-culturing type II *T. gondii*-infected THP-1 human monocytes with the brain cell lines inhibited the IDO1-mediated anti-*T. gondii* response in a GRA15-dependent manner. These data suggest that a GRA15-dependent virulence mechanism antagonizes the IDO1-dependent host immune response in human brain cells.

## Introduction

*Toxoplasma gondii* is a widespread protozoan that can infect most warm-blooded vertebrates. Infection with *T. gondii* causes toxoplasmosis in humans and animals (Boothroyd, 2009; Dubey, 2010). Nearly one-third of the human population is estimated to be infected with *T. gondii*. Although *T. gondii* infections in healthy individuals remain mostly asymptomatic, immunocompromised individuals often experience damage to their liver, brain, eyes, and other organs, thus resulting in lethal toxoplasmosis (Weitberg et al., 1979; Frenkel and Remington, 1980). In addition, *T. gondii* infections potentially lead to congenital toxoplasmosis in fetuses and newborn children via their primarily infected pregnant mothers (Montoya and Remington, 2008). Furthermore, the World Health Organization (WHO) and the Food and Agriculture Organization (FAO) have recently established toxoplasmosis as a foodborne infection of global concern (FAO/WHO, 2014). Thus, *T. gondii* is a common and important zoonotic pathogen.

Interferon-γ (IFN-γ) and the subsequent induction of IFN-stimulated genes (ISGs) are essential in anti-*T. gondii* host immune responses. Among ISGs, IFN-γ-inducible GTPases, such as p65 guanylate-binding proteins (GBPs), and p47 immunity-related GTPases (IRGs), have been shown to be important for clearing *T. gondii* in mice (Yamamoto et al., 2009; Gazzinelli et al., 2014). In addition, inducible nitric oxide synthase (iNOS) plays an important role in suppressing *T. gondii* growth in mice (Scharton-Kersten et al., 1997). In human cells, IFN-γ-inducible indoleamine 2,3-dioxygenase 1 (IDO1), rather than IFN-γ-inducible GTPases, and iNOS, is reported to play a major role in inhibiting *T. gondii* growth by degrading tryptophan, which is an essential amino acid for intracellular parasitic growth (Pfefferkorn et al., 1986a,b) in many human cell types (Bando et al., 2018b).

When *T. gondii* infects host cells, various effector molecules are secreted from dense granules to resist the IFN-γ-induced antiparasitic host immune responses in the human cells (Hunter and Sibley, 2012). A *T. gondii* dense granule protein TgIST directly inhibits STAT1-mediated IDO1 expression (Rosowski et al., 2014; Olias et al., 2016; Bando et al., 2018b). In addition, we recently found that another *T. gondii* dense granule protein GRA15 indirectly inhibits IDO1-dependent anti-*T. gondii* responses in human hepatocytes co-cultured with monocytes (Bando et al., 2018a). In detail, *T. gondii*-infected monocytes secrete interleukin-1β (IL-1β) in a GRA15-dependent manner. Subsequently, the secreted IL-1β mediates iNOS expression and nitric oxide (NO) production in IFN-γ-stimulated hepatocytes. Because iNOS and NO are strong negative regulators of IDO1, NO reduces IDO1 mRNA, and protein levels (Nathan and Xie, 1994; Thomas et al., 1994). Thus, *T. gondii* can proliferate in co-cultures of monocytes and hepatocytes in a GRA15-dependent manner. Because the GRA15-dependent virulence mechanism relies on iNOS induction in human hepatocytes in response to IL-1β and IFN-γ, other human cell types that can induce iNOS in response to IL-1β may allow GRA15-dependent *T. gondii* proliferation. However, which cell types are sensitive to GRA15-dependent functions when co-cultured with human monocytes remains unclear.

In the present study, we found iNOS-dependent IDO1 degradation in human brain cell lines (A172, IMR-32, and T98G) and human primary neurons. We further showed that GRA15 effectors play key roles in proparasitic functions in human brain cells when co-cultured with type II *T. gondii*-infected monocytes. These data demonstrate that *T. gondii* uses a GRA15-dependent virulence mechanism to suppress the IDO1-dependent anti-*T. gondii* immune responses in brain cells and hepatocytes.

## Results

### IL-1β Stimulation in Human Brain Cells Downregulates IDO1 mRNA Expression Levels

**Figure 1:**
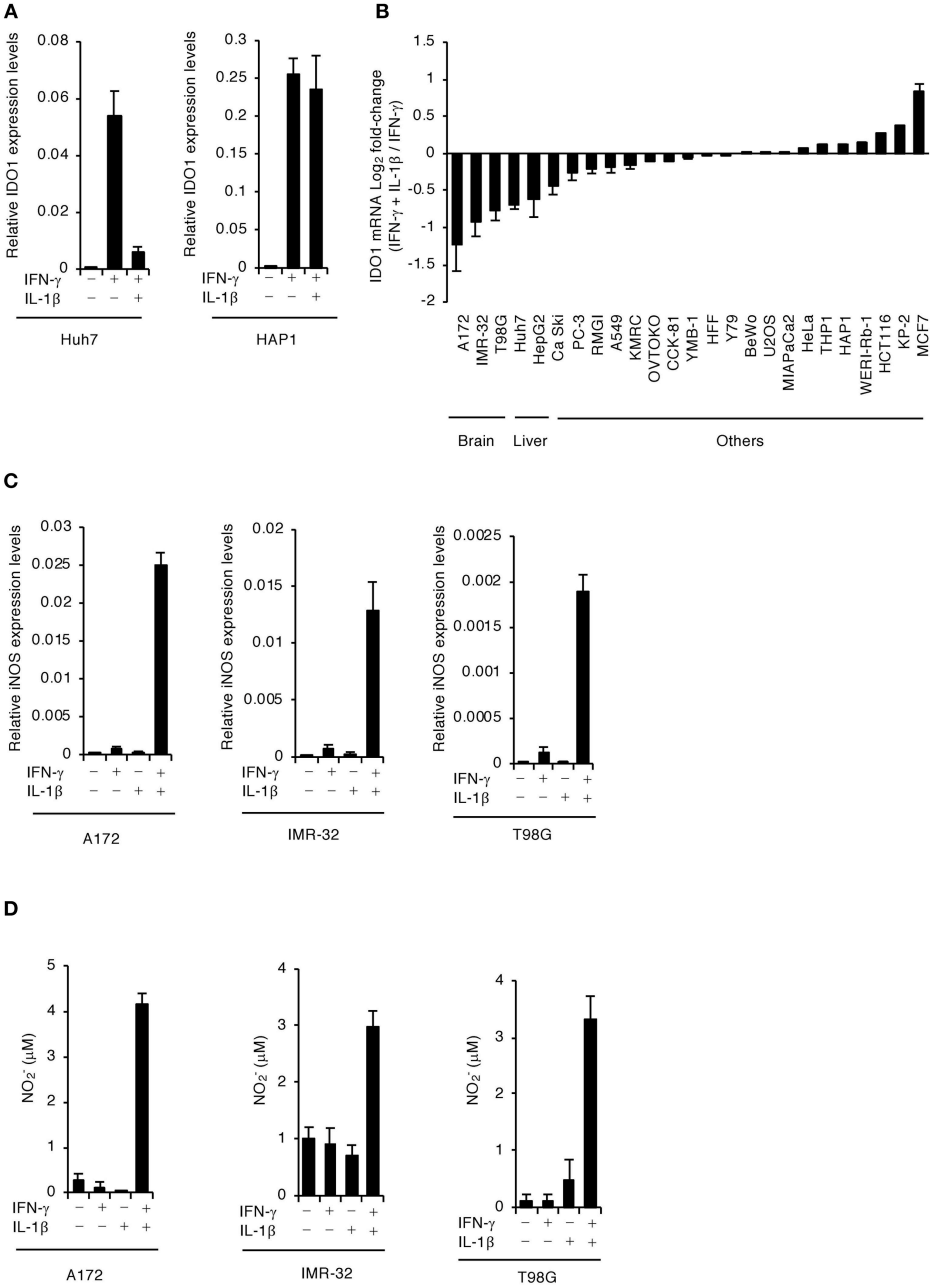
IFN-γ and IL-1β together induce iNOS expression and NO production in human brain cell lines. **(A)** Quantitative RT-PCR analysis of IDO1 mRNA level in IFN-γ- and/or IL-1β-stimulated Huh7 or HAP1 cells for 24 h. **(B)** Quantitative RT-PCR analysis of IDO1 mRNA level in various cell types upon stimulation with both IFN-γ and IL-1β as compared to IFN-γ stimulation. **(C)** Quantitative RT-PCR analysis of iNOS mRNA level in IFN-γ- and/or IL-1β-stimulated the brain cell lines (A172, IMR-32, or T98G). **(D)** Level of NO_2_ released into the culture supernatant of the brain cell lines (A172, IMR-32, or T98G) was measured by ELISA. Indicated values are means of ± s.d. (three biological replicates per group from three independent experiments) **(A–D)**.

We previously showed that costimulating IFN-γ and IL-1β significantly reduced IDO1 mRNA expression in the human hepatocyte cell line, Huh7 (Bando et al., 2018a), but not in the leukocyte cell line, HAP1 (Figure 1A). To assess whether IL-1β-dependent reduction of IDO1 mRNA is specific to hepatocytes or occurs in other human cell types, we first tested the effect of IL-1β stimulation on downregulation of IFN-γ-induced IDO1 mRNA expression levels in various human cell types (Figure 1B). We confirmed that IL-1β stimulation in liver cell lines (Huh7 and HepG2) strongly suppressed the IFN-γ-induced IDO1 mRNA expression levels (Figure 1B). Interestingly, IL-1β stimulation in all tested brain cell lines (A172; glioblastoma, IMR-32; neuroblastoma, and T98G; glioblastoma) severely reduced IFN-γ-induced IDO1 mRNA expression levels in a manner similar to or greater than that of the liver cell lines (Figure 1B). In contrast, adding IL-1β had either no or a lesser suppressive effect on IFN-γ-induced IDO1 mRNA expression levels in colonic (HCT116, CCK-81), leukocytic (HAP1, THP-1), lung (A549, PC-3), breast (MCF7, YMB-1), pancreatic (MIA PaCa-2, KP-2), kidney (293T, KMRC-1), ovarian (RMG-I, RKN), placental (BeWo), splenic (OVTOKO), cervical (Ca Ski, HeLa), osteosarcoma (U2OS), foreskin fibroblastic (HFF), and retinoblastic (Y79, WERI-Rb-1) cell lines (Figure 1B). These results suggest that IL-1β regulation reduces IDO1 mRNA in human brain cells as well as hepatocytes.

### Costimulating IFN-γ and IL-1β in Human Brain Cells Induces iNOS mRNA and NO

NO induced by iNOS expression downregulates IDO1 expression transcriptionally, translationally, and posttranslationally (Alberati-Giani et al., 1997; Daubener et al., 1999). In addition, we confirmed that costimulating IFN-γ and IL-1β significantly induced iNOS mRNA expression and NO production in Huh7 (Figure S1) as previously reported (Bando et al., 2018a). We found that IFN-γ stimulation alone did not induce iNOS mRNA expression or NO production in A172 glioblastoma, IMR-32 neuroblastoma, or T98G glioblastoma human brain cell lines (Figures 1C,D). Conversely, stimulating both IL-1β and IFN-γ strongly induced iNOS mRNA expression and NO production in all brain cell lines tested (Figures 1C,D). These results suggest that costimulating IL-1β and IFN-γ leads to iNOS expression and NO production in human brain cells.

### IL-1β Stimulation in Human Brain Cells Downregulates IDO1-Dependent Anti-*T. gondii* Immune Responses

**Figure 2:**
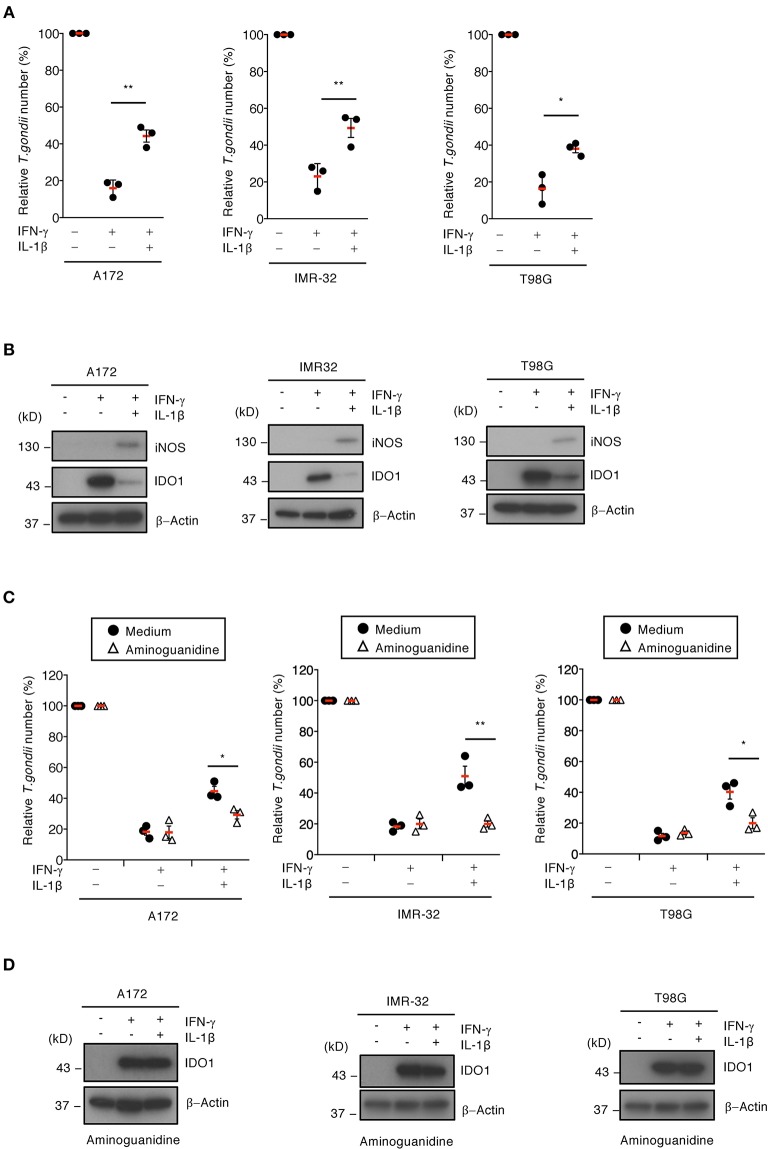
iNOS suppresses IDO1-dependent anti-*T. gondii* response in human brain cell lines. **(A)** Luciferase assay of the parasite survival rate at 24 h post infection in IFN-γ- and/or IL-1β-stimulated the brain cell lines (A172, IMR-32, or T98G). **(B)** Western blot analysis showing the expression of iNOS, IDO1, and β-Actin in the indicated cells after stimulation with IFN-γ and/or IL-1β for 24 h. **(C)** Luciferase assay of the parasite survival rate at 24 h post infection in the cells treated with the indicated cytokines and/or aminoguanidine. **(D)** Western blot analysis showing the expression of IDO1 and β-Actin in the presence of aminoguanidine. Each western blot image is representative of three independent experiments **(B,D)**. Indicated values are means of ± s.d. (three biological replicates per group from three independent experiments) **(A,C)**. ^*^p < 0.05; ^**^p < 0.01, (Student's *t*-test).

We previously showed that IFN-γ-induced IDO1 plays a major role in anti-*T. gondii* responses in various human cells (Bando et al., 2018b). To examine whether IL-1β-induced iNOS expression and NO production are involved in proparasitic functions in A172 glioblastoma, IMR-32 neuroblastoma, and T98G glioblastoma cells, we compared the IFN-γ-mediated reduction of *T. gondii* numbers in the presence or absence of IL-1β (Figure 2A). IFN-γ alone strongly suppressed *T. gondii* numbers in all brain cell lines tested (Figure 2A). In contrast, costimulating IFN-γ and IL-1β impaired the IFN-γ-mediated anti-*T. gondii* responses in all brain cell lines tested (Figure 2A). Next, we examined IDO1 and iNOS protein levels in the presence or absence of IL-1β (Figure 2B). IFN-γ stimulation alone resulted in high IDO1 expression levels, while iNOS protein expression was undetected in A172 glioblastoma, IMR-32 neuroblastoma, and T98G glioblastoma cells (Figure 2B). In contrast, costimulating IFN-γ and IL-1β induced iNOS protein expression and conversely reduced IDO1 protein expression levels (Figure 2B). Next, to test whether iNOS expression is important for IL-1β-dependent IDO1 reduction, we assessed the effect of the selective iNOS inhibitor, aminoguanidine, on proparasitic functions (Figure 2C). *T. gondii* numbers were significantly reduced in the presence of aminoguanidine (Figure 2C) in the brain cell lines tested. In addition, aminoguanidine treatment inhibited NO production (Figure S2) and prevented IDO1 protein level reductions in all IFN-γ- and IL-1β-costimulated A172 glioblastoma, IMR-32 neuroblastoma, and T98G glioblastoma human brain cell lines (Figure 2D). These data suggest that iNOS expression and NO production are important for IL-1β-dependent proparasitic functions in human brain cells.

### iNOS Is Critical for GRA15-Dependent Proparasitic Functions in Human Brain Cells Co-cultured With Monocytes

**Figure 3:**
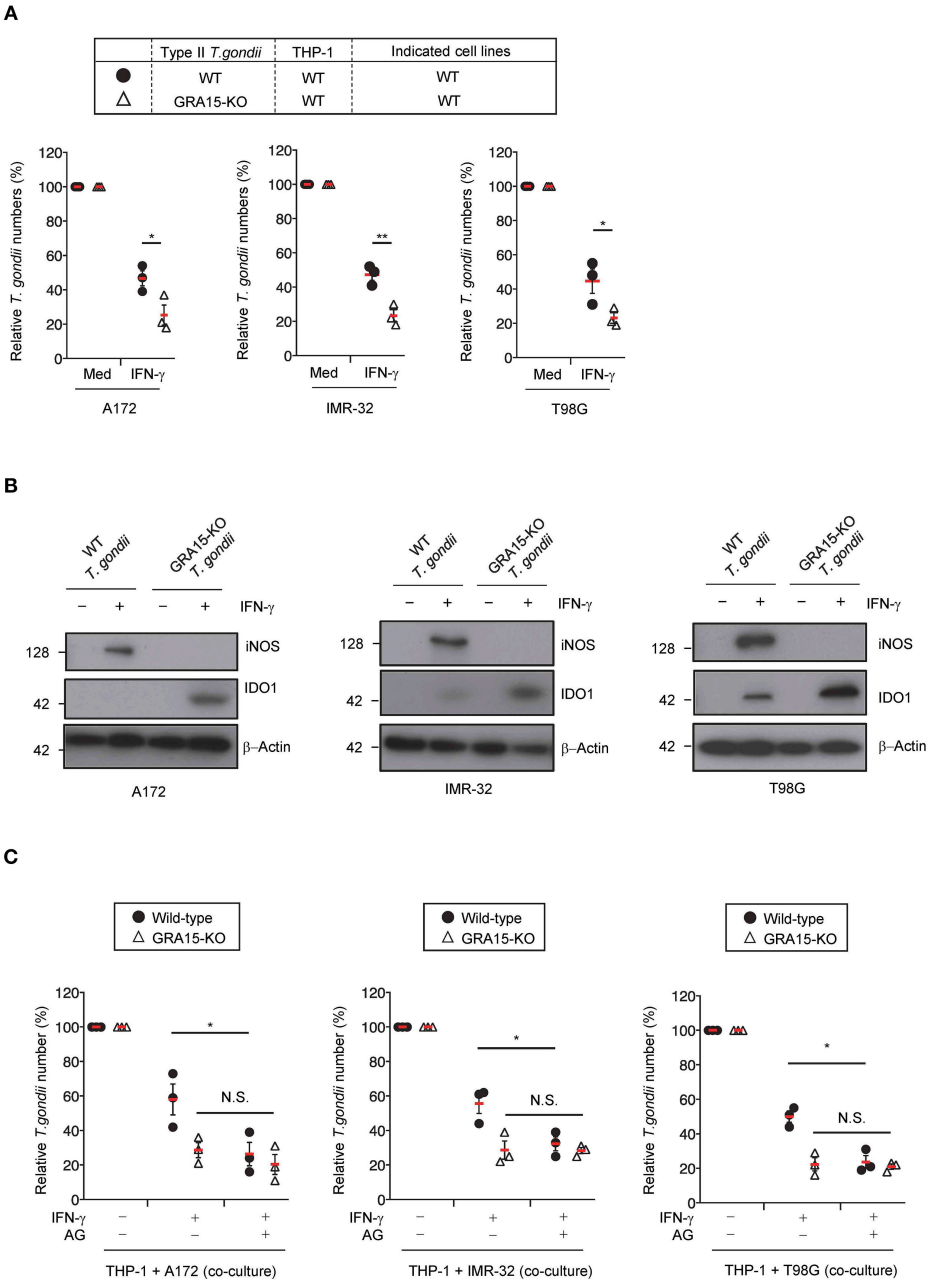
GRA15 inhibits IFN-γ-dependent anti-*T. gondii* response in co-culture conditions of THP-1 and human brain cell lines. **(A,B)** THP-1 cells were infected with WT or GRA15-KO Pru *T. gondii*. The infected THP-1 cells were co-cultured with A172, IMR-32, or T98G cells in the presence or absence of IFN-γ for 48 h. **(A)** Luciferase assay of the parasite survival rate. **(B)** Western blot analysis showing the expression of iNOS, IDO1, and β-Actin in the indicated cells. **(C)** THP-1 cells were infected with WT or GRA15-KO Pru *T. gondii*. The infected THP-1 cells were co-cultured with A172, IMR-32, or T98G cells and then left untreated or treated with IFN- and/or aminoguanidine. In the presence or absence of IFN-γ for 48 h. The parasite survival rates were measured by luciferase assay. Each western blot image is representative of three independent experiments **(B)**. Indicated values are means of ± s.d. (three biological replicates per group from three independent experiments) **(A,C)**. ^*^p < 0.05; ^**^p < 0.01, N.S., not significant; (Student's *t*-test).

We previously showed that *Toxoplasma* effector GRA15 has a virulence function in co-cultures of the human monocyte cell line, THP-1, and the human hepatocyte cell line, Huh7 (Bando et al., 2018a). Next, we examined whether GRA15 is involved in proparasitic functions in human brain cell lines co-cultured with THP-1 cells. A172 glioblastoma, IMR-32 neuroblastoma, and T98G glioblastoma cell lines produced NO when co-cultured with wild-type Pru *T. gondii* type II strain-infected THP-1 cells (Figure 3A). In contrast, the brain cell lines co-cultured with GRA15-knockout (KO) Pru *T. gondii*-infected THP-1 cells did not produce NO (Figure S3A). We next assessed parasite numbers in wild-type or GRA15-KO Pru *T. gondii*-infected THP-1 cells co-cultured with A172 glioblastoma, IMR-32 neuroblastoma, and T98G glioblastoma cell lines (Figure 3A). Parasite numbers in the GRA15-KO Pru *T. gondii* co-cultures with human brain cell lines and THP-1 cells were significantly lower than those that included wild-type Pru *T. gondii*-infected THP-1 (Figure 3A). Furthermore, iNOS protein expression and IDO1 protein reduction occurred in co-cultures with THP-1 cells infected with wild-type Pru *T. gondii* but not GRA15-KO *T. gondii* (Figure 3B). On the other hand, survival of wild-type *T. gondii* infecting neuronal cell lines alone was comparable to that of GRA15-KO *T. gondii* (Figure S3B). Next we examine whether iNOS inhibition restores GRA15-dependent reduction of parasite growth in wild-type *T. gondii* infected co-cultured conditions (Figure 3C). Aminoguanidine treatment significantly reduced numbers of wild-type *T. gondii* but not those of GRA15-KO parasites (Figure 3C). These results indicate that GRA15 is specifically required for iNOS-dependent IDO1 reduction and parasitic growth in human brain cell line and THP-1 cell co-cultures in the presence of IFN-γ.

### Strain-Specific IL-1β-Induced IDO1 Suppression Facilitates *T. gondii* Survival

**Figure 4:**
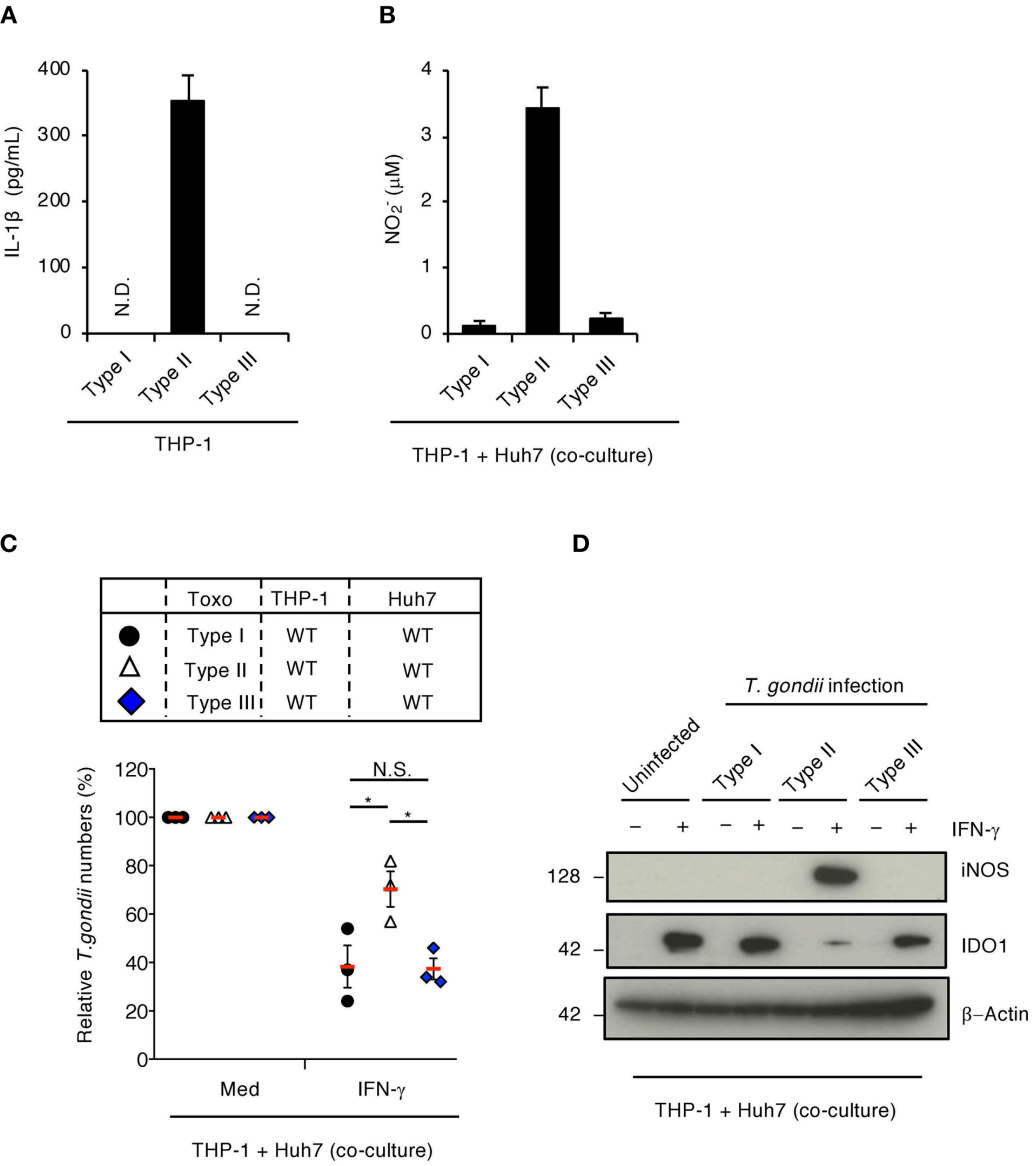
Strain-specific inhibition of IFN-γ-dependent anti-*T. gondii* response in co-culture conditions of THP-1 and Huh7 cells. **(A)** THP-1 cells were infected with type I (RH), type II (Pru), or type III (CTG) *T. gondii* for 24 h. Level of IL-1β released into the culture supernatant was measured by ELISA. **(B,C)** The infected THP-1 cells with different *T. gondii* strain were co-cultured with Huh7 cells in the presence or absence of IFN-γ for 48 h. **(B)** Level of NO_2_ released into the culture supernatant was measured by ELISA. **(C)** Luciferase assay of the parasite survival rate. Indicated values are means of ± s.d. (three biological replicates per group from three independent experiments) **(A–C)**. ^*^p < 0.05; (Student's *t*-test).

GRA15 is involved in sustained NF-κB activation in cells infected with the type II *T. gondii* strain, but not the type I or type III strains (Gov et al., 2013). In addition, GRA15's amino acid sequence varies among *T. gondii* strains (Rosowski et al., 2011; Gay et al., 2016). Therefore, we tested whether the GRA15-mediated virulence mechanism is strain-specific. When IL-1β production was tested in THP-1 cells, infection with the type II *T. gondii* strain, but not the type I or type III strains, induced IL-1β production in THP-1 cells (Figure 4A) as previously reported (Gov et al., 2013). In addition, NO was produced when THP-1 cells were infected with the type II strain, but not the type I or type III strains, when co-cultured with Huh7 cells (Figure 4B). Moreover, GRA15-dependent proparasitic functions were observed with the type II strain infection but not the type I or type III strain infections (Figure 4C). Furthermore, iNOS protein expression and IDO1 protein reduction occurred in co-cultures with THP-1 cells infected type II *T. gondii* strains, but not the type I or type III strains (Figure 4D) These results suggest that type II GRA15-mediated virulence mechanisms are strain-specific under co-culture conditions.

### Type II GRA15-Dependent IDO1 Reduction by iNOS-Mediated NO in Co-cultures of Primary Human Monocytes and Neurons

**Figure 5:**
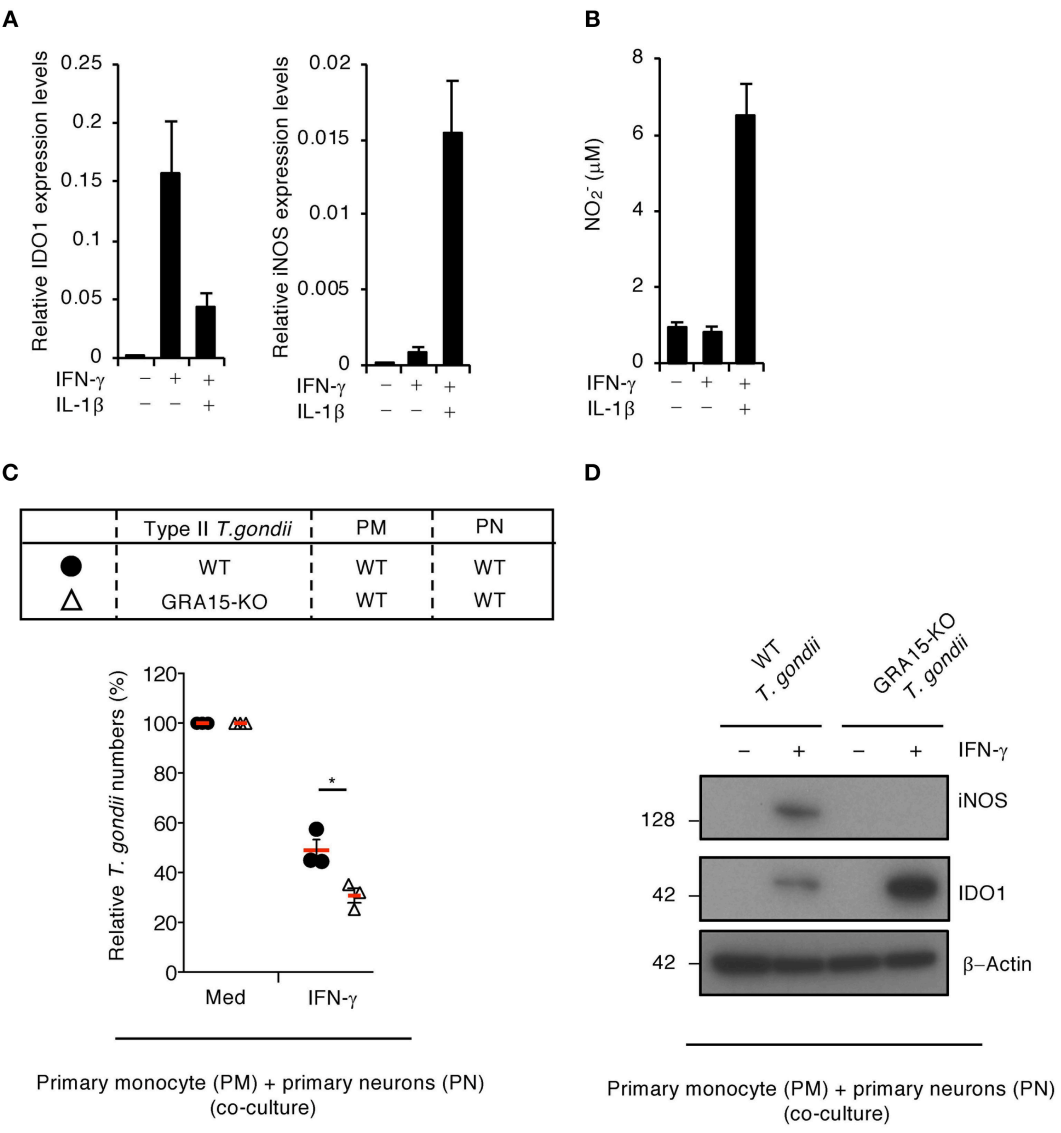
GRA15-dependent virulence mechanism is functional in primary neurons when co-cultured with primary monocytes. **(A,B)** Primary human neurons were either untreated or treated with the indicated cytokines for 24 h. **(A)** Quantitative RT-PCR analysis of the mRNA level of IDO1 and iNOS in the cells. **(B)** Level of NO_2_ released into the culture supernatant was measured by ELISA. **(C,D)** Primary human monocytes were infected with WT or GRA15-KO Pru *T. gondii* for 24 h. The infected monocytes were co-cultured with primary human neurons in the presence or absence of IFN-γ for 48 h. **(C)** Luciferase assay of the parasite survival rate. **(D)** Western blot analysis showing the expression of iNOS, IDO1, and β-Actin. Each Western blot image is representative of three independent experiments **(D)**. Indicated values are means of ± s.d. (three biological replicates per group from three independent experiments) **(A–C)**. ^*^p < 0.05; (Student's *t*-test).

To examine whether type II GRA15-dependent proparasitic functions exist in primary human neurons, we tested the IDO1 and iNOS mRNA expression levels (Figure 5A) as well as NO production (Figure 5B) in the presence or absence of IL-1β in primary human neurons. IFN-γ alone stimulated high IDO1 expression levels, whereas iNOS protein expression and NO production were undetected (Figures 5A,B). In contrast, IFN-γ and IL-1β costimulation led to iNOS protein expression and NO production and conversely reduced IDO1 protein levels (Figures 5A,B). Next, to assess whether type II GRA15-dependent proparasitic functions exist in primary human neurons when co-cultured with primary human monocytes, we compared the number of wild-type or GRA15-KO Pru *T. gondii* in these co-cultures (Figure 5C). The number of GRA15-KO parasites in the co-cultures was significantly lower than the number of wild-type parasites (Figure 5C). In addition, iNOS expression, NO production and IDO1 reduction were observed in the co-cultures containing wild-type Pru *T. gondii* but not in those containing GRA15-KO parasites (Figures 5D and Figure S3C). These results indicate that type II *Toxoplasma* effector type II GRA15-dependent virulence mechanisms operate in co-cultures of human primary neurons and human primary monocytes and are required for counter defense against IFN-γ-induced IDO1-dependent anti-*T. gondii* responses in humans.

## Discussion

We previously showed that IL-1β stimulation inhibits IFN-γ-induced IDO1 mRNA expression in the hepatocyte cell line, Huh7, and primary human hepatocytes (Bando et al., 2018a). In this study, we found that IL-1β also inhibits IDO1 mRNA expression in the A172 glioblastoma, IMR-32 neuroblastoma, and T98G glioblastoma human brain cell lines and in primary human neurons. Interestingly, the 5 cell lines, Huh7 hepatoma, HepG2 hepatoma, A172 glioblastoma, IMR-32 neuroblastoma, and T98G glioblastoma cell lines, which are derived from human liver and brain tissue, were ranked in the top 5 for IL-1β-dependent IDO1 reduction, suggesting that IL-1β suppresses IDO1-dependent host immunity in the human brain and liver.

*T. gondii* infects its host by using migratory immune cells, such as neutrophils, dendritic cells (DCs), and monocytes, to spread throughout the body via a mechanism known as the Trojan horse mechanism (Coombes et al., 2013). This enables infected immune cells to make contact with various cells, tissues, and organs. Although *T. gondii* can potentially infect all nucleated cells, the parasite is often isolated from specific organs such as the liver and brain (Robert-Gangneux and Darde, 2012). Here, we found that the *Toxoplasma* effector GRA15-dependent virulence mechanism operates in human liver and brain cells. Our findings suggest that GRA15-dependent virulent mechanisms may define the liver and brain specificity. We previously reported that IDO1 plays a major role in anti-*T. gondii* responses in various human cells (Bando et al., 2018b), whereas IDO1-independent anti-*T. gondii* immune responses involve ATG16L1 and GBP1 in some human cell lines (Selleck et al., 2015; Clough et al., 2016). However, the GRA15 virulence mechanism requires secondarily infected cells to express iNOS in response to IFN-γ and IL-1β, thus reducing IDO1 mRNA, and protein expressions. Therefore, although an IDO1-dependent anti-*T. gondii* response is observed in various cells, the GRA15-dependent virulence mechanism targeting IDO1 operates in specific cells such as neurons and hepatocytes. Moreover, we have shown that the GRA15-dependent virulence mechanism to suppress IDO1-mediated anti-*T. gondii* immune response requires IL-1β production in the primarily infected monocytes and subsequent iNOS expression in the secondarily infected hepatocytes in the previous study (Bando et al., 2018a) or brain cells in this study. In the co-culture of monocytes and hepatocytes or neurons, IL-1β derived from *T. gondii*-infected monocytes in a manner dependent on GRA15 stimulates hepatocytic or neuronal production of iNOS and NO in the presence of IFN-γ, resulting in suppression of IDO1 mRNA and protein expressions. On the other hand, since there is no source of IL-1β, which is essential for neuronal induction of iNOS, in the single culture, the brain cells can robustly induce IDO1, and remain the expression levels, enabling suppression of *T. gondii* growth. Taken together, presence of IL-1β from *T. gondii*-infected monocytes characterizes the GRA15-dependent virulence mechanism that is observable in the co-culture system but not in the single culture of brain cells. Although we found a virulent role of GRA15 to suppress immune response in the co-culture of human monocytes and neurons is found in this study, we failed to find such an immune-suppressive function in the single culture of neurons. Whether GRA15 possesses a unique function in neurons alone will be assessed in the future.

In the present study, we demonstrated that primary neurons exhibit the GRA15-dependent iNOS expression and IDO1 reduction in a manner similar to various neuronal cell lines that tend to be considered to be distinct from physiological neurons and therefore underestimated as fibroblasts with neuronal cell markers such as GFAP. Given neuronal cell lines are materially inexhaustible and can be genetically modified, they might be relatively more useful than primary neurons for further investigation of the detailed molecular mechanisms of GRA15-dependent *T. gondii* virulence in humans.

*T. gondii* is classified into three major clonal lineages known as types I, II, and III (Howe and Sibley, 1995a; Darde, 1996). Type I is a highly virulent strain in mice, while type II exerts intermediate virulence, and type III exerts low virulence. The current study demonstrates that type II *T. gondii* strain can survive more than types I or III parasites in co-culture of human neuronal cells and monocytes, suggesting that *T. gondii* virulence might be different between humans and mice, probably among host species. Notably, type II *T. gondii* is the most prevalent cause of both human congenital and acquired toxoplasmosis in North America and Europe (Howe and Sibley, 1995b; Ajzenberg et al., 2002, 2009). Our previous and current findings involving GRA15 can be one of the mechanisms that might account for *T. gondii* tropism in human brains and livers. However, the co-culture system in the previous and current studies have observed for only 48 h that is insufficient to assess bradizoite transformation and cyst formation. Obviously, further investigations to examine whether the presence of type II GRA15 affects bradyzoite transformation in the co-culture system may be of interest in the future.

In summary, we demonstrated that the *T. gondii* effector, GRA15, plays an important role in inhibiting IFN-γ-inducible IDO1-dependent anti-*T. gondii* responses in human brain and liver cells when co-cultured with human monocytes. Because GRA15-dependent virulence mechanisms are important for *T. gondii* infections in humans, further elucidation may contribute to developing advanced therapeutic strategies to treat human toxoplasmosis.

## Materials and Methods

### Cells and Parasites

All *T. gondii* strains (type I; RH strain, type II; Pru strain, type III; CTG strain) were maintained in Vero cells in RPMI (Nacalai Tesque) supplemented with 2% heat-inactivated FBS (JRH Bioscience), 100 U/mL penicillin/streptomycin (Nacalai Tesque), as previously described (Ma et al., 2014). GRA15-deficient Pru *T. gondii* was generated our previous study (Bando et al., 2018a). HAP1 (myelogenous leukemia) cells were maintained in IMDM (Nacalai Tesque) containing 10% heat-inactivated FBS, 100 U/mL penicillin/streptomycin. BeWo (choriocarcinoma), PC-3 (lung adeno carcinoma), HepG2 (hepatoma), OVTOKO (ovarian carcinoma), YMB-1 (breast carcinoma), KP-2 (tubular adenocarcinoma), THP-1 (acute monocytic leukemia), WERI-Rb-1 (Retinoblastoma), RKN (leiomyosarcoma), Ca Ski (epidermoid carcinoma), Y79 (Retinoblastoma), HFFs (fibroblast), and Huh7 (hepato cellular carcinoma) cells were maintained in RPMI (Nacalai Tesque) containing 10% heat-inactivated FBS, 100 U/mL penicillin/streptomycin. A172 (glioblastoma), T98G (glioblastoma), IMR-32 (neuroblastoma), CCK-81 (adenocarcinoma), MCF-7 (breast adenocarcinoma), KMRC-1 (renal carcinoma), A549 (lung carcinoma), U2OS (bone osteosarcoma), RMG-I (Ovarian mesonephroid adenocarcinoma), MIA PaCa-2 (pancreatic cancer), and HeLa (cervix epitheloid carcinoma) cells were maintained in DMEM (Nacalai Tesque) containing 10% heat-inactivated FBS, 100 U/mL penicillin/streptomycin. Cryopreserved primary human monocytes (c-12909) were obtained from TAKARA. Cryopreserved primary human neurons (#1520) were obtained from ScienCell. According to the manufacture, the primary human neurons are cortical neurons isolated from embryonic tissue. The neurons are characterized by neurofilament, MAP2, and β-tubulin III. Primary cells were maintained in neuronal medium (ScienCell).

### Reagents

Rabbit anti-IDO1 polyclonal antibody (13268-1-AP) was obtained from Proteintech. Mouse anti-iNOS monoclonal antibody (NOS2; sc-7271) was obtained from Santa Cruz Biotechnology. Mouse anti-β-Actin monoclonal antibody (A1978) was obtained from Sigma. Recombinant human IFN-γ and IL-1β were obtained from Peprotech. Aminoguanidine hydrochloride (396494) was obtained from Sigma. Purified anti-human IL-1β (511601) and biotin anti-human IL-1β antibodies (511703) were obtained from BioLegend.

### Quantitative RT-PCR

Total RNA was extracted, and cDNA was synthesized using the Verso Reverse transcription kit (Thermo Fisher Scientic). Quantitative RT-PCR was performed with a CFX connect real-time PCR system (Bio-Rad Laboratories) using the Go-Taq Real-Time PCR system (Promega). The values were normalized to the amount of glyceraldehyde 3-phosphate dehydrogenase (GAPDH) for human cells. Sequences of all primers are listed in Table S1.

### Western Blot Analysis

Cells were lysed in a lysis buffer (0.5% Nonidet P-40, 150 mM NaCl, and 20 mM Tris-HCl, pH 7.5) containing a cocktail of protease inhibitors (Roche). The cell lysates were separated by SDS-PAGE and transferred to polyvinylidene difluoride membranes (Immobilon-P, Millipore). Western blot analysis was performed using the indicated antibodies as described previously in detail (Yamamoto et al., 2009).

### Luciferase Assay

Luciferase activities of total cell lysates were measured using standard protocols as described previously (Yamamoto et al., 2009). Cells were untreated or treated with 10 ng/mL IFN-γ and/or 20 ng/mL IL-1β before 24 h or at the same time of the luciferase-expressing *T. gondii* infection (MOI = 0.5–1). To measure the number of *T. gondii*, all infected cells were collected for the indicated periods and lysed by 100 μl of lysis buffer (Promega), followed by sonication. After centrifugation at 20,000 × g at 4°C, the luciferase activity of the supernatant was measured using the Dual Luciferase Reporter Assay System (Promega) and a GLOMAX 20/20 luminometer (Promega). The percentages of the luciferase activities in cytokines-stimulated cells compared to those in unstimulated cells were shown as “Relative *T. gondii* numbers” in figures.

### Measurement of the Production of NO_2_

5 × 10^5^ A172, T98G, Huh7 cells, or 1 × 10^6^ IMR-32 or HAP1 were cultured in 12- or 24-well plates with 10 ng/mL IFN-γ and/or 10 ng/mL IL-1β for the indicated periods. The concentration of NO in the culture supernatant was measured using a NO_2_/NO_3_ Assay Kit-FX (Dojindo).

### Stimulation of Cell Lines With IFN-γ and IL-1β

1 × 10^6^ HAP1, PC-3, HepG2, YMB-1, THP-1, IMR-32, RMG-I, Ca Ski, or CCK-81, or 5 × 10^5^ BeWo, OVTOKO, KP-2, WERI-Rb-1, RKN, HFFs, Huh7, A172, U2OS, T98G, MCF-7, HeLa, HCT116, KMRC-1, MIA PaCa-2, A549, Y79 cells were untreated or treated with 10 ng/mL IFN-γ and/or 20 ng/mL IL-1β for 24 h. Then, untreated or treated cells were used for quantitative RT-PCR, Western blot or parasite infection.

### Infection of the Cell Lines With *T. gondii* in Single-Culture System

5 × 10^5^ A172, T98G, or 1 × 10^6^ IMR-32 were untreated or treated with 10 ng/mL IFN-γ and/or 20 ng/mL IL-1β for 24 h, then the luciferase-expressing *T. gondii* were infected (MOI = 0.5). At 24 h after parasite infection, the cell lysates and cell culture supernatants were collected and used for each experiment. Luciferase activities of total cell lysates in the single-culture condition were measured.

### Infection of the Cell Lines With *T. gondii* in Co-culture System

5 × 10^5^ A172, T98G, 1 × 10^6^ IMR-32, or 1 × 10^5^ primary human neurons were cultured in 12- or 24-well plates for 24 h and washed twice with PBS before co-culture. 5 × 10^4^ Primary human monocytes or 2.5 × 10^5^ or 5 × 10^5^ THP-1 cells were plated in 24-well plates and infected or uninfected with the luciferase-expressing *T. gondii* (MOI = 1). At 24 h post infection, the cell culture supernatants containing either uninfected or infected primary human monocytes or THP-1 cells were added directly on top of the 24-well plates containing the human brain cell lines or primary human neurons in the presence or absence of 10 ng/mL IFN-γ. At 48 h after treatment with IFN-γ, the cell lysates, and cell culture supernatants were collected and used for each experiment. Luciferase activities of total cell lysates in the co-culture condition were measured.

### Inhibitor Treatment

5 × 10^5^ A172 or T98G and 1 × 10^6^ IMR-32 were treated with 10 ng/mL IFN-γ and/or aminoguanidine hydrochloride (500 μM) for 24 h and then infected or uninfected with *T. gondii* as described above.

### Statistical Analysis

All statistical analyses were performed using Graphpad Prism 7 (GraphPad Software) or Excel (Microsoft). All the experimental data represent the average of three biological replicates (three independent experiments). The statistical significance of differences in mean values was analyzed by using an unpaired two-tailed Student's *t*-test. *p* < 0.05 were considered to be statistically significant.

## Author Contributions

HB and MY designed the study. HB, YL, NS, AP, RS, and ST performed the experiments. HB, JM, MS, and MY analyzed the data. HB, YL, and MY wrote the paper.

### Conflict of Interest Statement

The authors declare that the research was conducted in the absence of any commercial or financial relationships that could be construed as a potential conflict of interest.

## Supplementary Material

Figure S1 IL-1β-induced iNOS expression and NO production in Huh7 cells. Huh7 cells were either untreated or treated with the indicated cytokines for 24 h. (left panel of the figure) Quantitative RT-PCR analysis of iNOS mRNA level in the cells. (right panel of the figure) Level of NO_2_ released into the culture supernatant was measured by ELISA. Indicated values are means of ± s.d. (three biological replicates per group from three independent experiments).

Figure S2 The effect of aminoguanidine on NO production in human brain cell lines. A172, IMR-32, or T98G cells were either untreated or treated with the indicated cytokines in the presence of aminoguanidine for 24 h. Level of NO_2_ released into the culture supernatant was measured by ELISA. Indicated values are means of ± s.d. (three biological replicates per group from three independent experiments).

Figure S3 NO production in co-culture conditions. **(A)** THP-1 cells were infected with WT or GRA15-KO Pru *T. gondii* for 24 h. The infected THP-1 cells were co-cultured with A172, IMR-32, or T98G cells in the presence or absence of IFN-γ for 48 h. Level of NO_2_ released into the culture supernatant was measured by ELISA. **(B)** A172, IMR-32, or T98G cells were left untreated or treated with IFN-γ for 24 h and then infected with wild-type or GRA15-KO Pru *T. gondii*. The parasite survival rates were measured by luciferase assay. **(C)** Primary human monocytes were infected with WT or GRA15-KO Pru *T. gondii* for 24 h. The infected monocytes were co-cultured with primary human neurons in the presence or absence of IFN-γ for 48 h. Level of NO_2_ released into the culture supernatant was measured by ELISA. Indicated values are means of ± s.d. (three biological replicates per group from three independent experiments) **(A–C)** ^*^*p* < 0.05; (Student's *t*-test).

Table S1 List of primers used in this study. The information for primer names, restriction enzymes, sequences and the resulting plasmids is shown.
