# Supplementary figures and images for: Toxoplasma Effector GRA15-Dependent Suppression of IFN-γ-Induced Antiparasitic Response in Human Neurons
Source: Front Cell Infect Microbiol. 2019 May 1;9:140. doi: 10.3389/fcimb.2019.00140 (PMC6504700; doi:10.3389/fcimb.2019.00140)

Figure S1\_Bando et al.

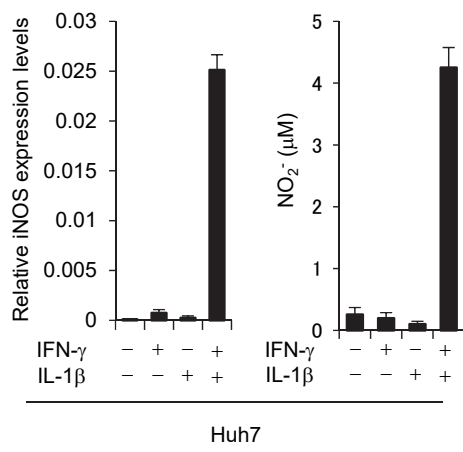

Figure S2\_Bando et al.

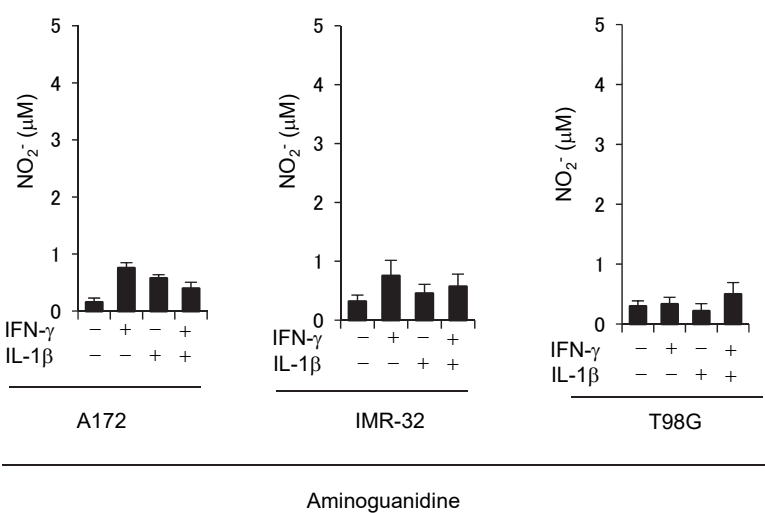

Figure S3\_Bando et al.

A

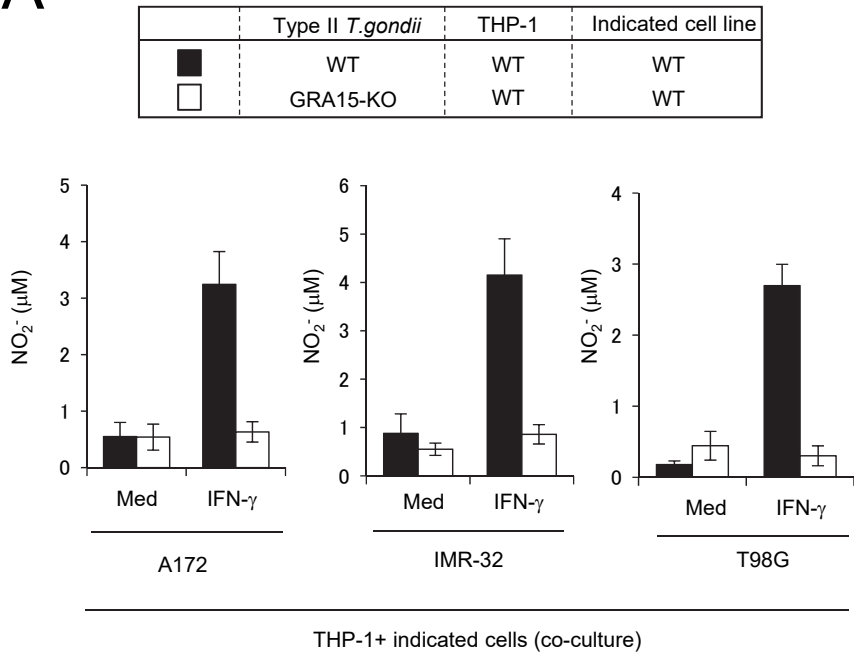

B

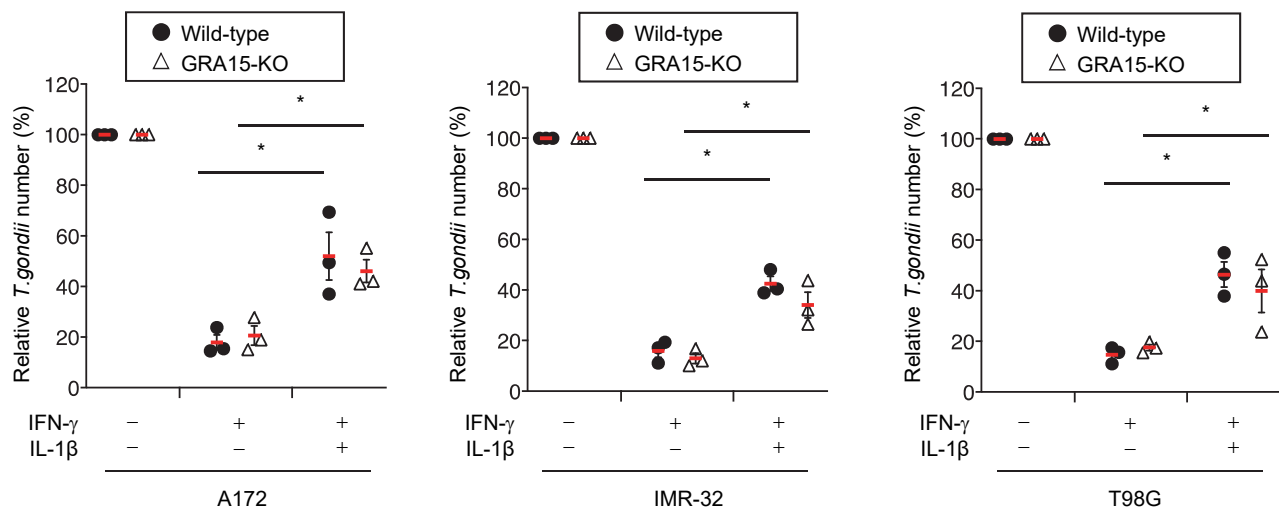

C

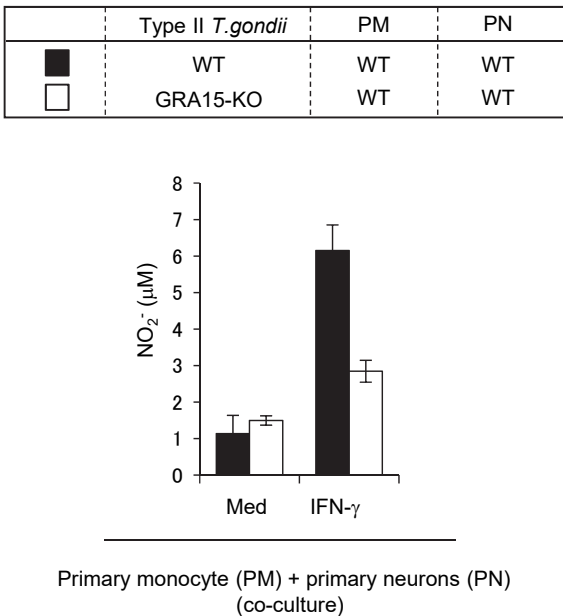

Supplement: Figure S1 — IL-1β-induced iNOS expression and NO production in Huh7 cells. Huh7 cells were either untreated or treated with the indicated cytokines for 24 h. (left panel of the figure) Quantitative RT-PCR analysis of iNOS mRNA level in the cells. (right panel of the figure) Level of NO2 released into the culture supernatant was measured by ELISA. Indicated values are means of ± s.d. (three biological replicates per group from three independent experiments). [file Data_Sheet_1.pdf]
