# Supplementary material for: Toxoplasma Effector GRA15-Dependent Suppression of IFN-γ-Induced Antiparasitic Response in Human Neurons
Source: Front Cell Infect Microbiol. 2019 May 1;9:140. doi: 10.3389/fcimb.2019.00140 (PMC6504700; doi:10.3389/fcimb.2019.00140)
Supplement: Table S1 — List of primers used in this study. The information for primer names, restriction enzymes, sequences and the resulting plasmids is shown. [file Data_Sheet_2.PDF]

Table S1_Bando et al.

TABLE S1.

Primer name	enzyme	sequence	Resulting plasmids and descriptions
hiNOS_F	—	5'-CACAGAGATCCACCTGACTGTGG-3'	Quantitative RT-PCR
hiNOS_R	—	5'-AAGCCGCTGGCATTCCGCACAAA-3'	Quantitative RT-PCR
hIDO1_F	—	5'-AGTGTTTCACCAAATCCACGATC-3'	Quantitative RT-PCR
hIDO1_R	—	5'-AAGCACTGAAAGACGCTGCTTTG-3'	Quantitative RT-PCR
hGAPDH_F	—	5'-GTCATGGGTGTGAACCATGAGAAG-3'	Quantitative RT-PCR
hGAPDH_R	—	5'-AGTCTTCTGGGTGGCAGTGATG-3'	Quantitative RT-PCR
